# Health Atlas: Tutorial of a Visualization Tool and Data Resource for Place-Based Social and Structural Determinants of Health

**DOI:** 10.2196/89065

**Published:** 2026-07-09

**Authors:** Debora L Oh, Kathryn E Kemper-McIsaac, Dan Meltzer, Eric Brelsford, Kelsey Taylor, Dhananjay Vinay Dixit, Andrea Nickerson, Riya Desai, Salma Shariff-Marco, Courtney Lyles, Scarlett Gomez, Mark J Pletcher, Mindy C DeRouen

**Affiliations:** 1 Department of Epidemiology and Biostatistics, University of California, San Francisco, 550 16th St., Floor 2, San Francisco, CA, 94143, United States, 1 415-476-2300; 2 Stamen Design, San Francisco, CA, United States; 3 Kaiser Permanente Oakland Medical Center, Oakland, CA, United States; 4 Center for Healthcare Policy and Research, University of California, Davis, Sacramento, CA, United States; 5 Department of Public Health Sciences, College of Health, Education, and Social Transformation, New Mexico State University, Las Cruces, NM, United States

**Keywords:** demography, socioeconomic factors, neighborhood characteristics, environment, social environment, public health, social determinants of health

## Abstract

Places have a significant impact on health. Thus, examining place-based structural and social determinants can help inform effective public health interventions. HealthAtlas.ucsf.edu provides a single online platform to explore multiple domains of place-based data. The initial California-only version of Health Atlas was launched in April 2020, and a national version was launched in October 2024. An artificial intelligence–assisted search function was integrated in January 2026. Health Atlas includes data on over 200 variables across 5 topical domains: demographics, socioeconomic, neighborhood, environment, and health and health care. The data were either (1) obtained from the American Community Survey, CDC PLACES, and other public sources or (2) obtained through collaboration with research partners. Users can visualize and aggregate data for 7 geographic levels: census tract, zip code tabulation area, county, congressional district, core-based statistical area, public use microdata area, and state. Most variables are available across all 50 states in the United States, the District of Columbia, and Puerto Rico. Users can explore Health Atlas to better understand the relationship between selected variables via maps, histograms, scatterplots, and summary plots. Users can select custom areas to suit specific needs. Customizable datasets can be downloaded for further use. Health Atlas provides freely available, user-friendly data and tools to support researchers, community organizations, government entities, and other public health professionals. We envision that Health Atlas will contribute to evidence-based, community-based initiatives; impactful health equity research; and effective public health programs.

## Introduction

When it comes to health, place matters. Where we live, work, and play influences health in both positive and negative ways. Places have a significant impact on health outcomes independent of individual-level factors [1]. Examining place-based social determinants of health, such as access to food, housing, air quality, and social support, as well as structural determinants, including the historical context, governance, policies, and norms that shape local environments, can help inform effective public health interventions [2,3].

While the concept of place can be subjective, smaller areas are often defined by census tract or zip code boundaries, and larger areas are often defined by counties, congressional districts, or states. Using these definitions, publicly accessible place-based data can be leveraged to identify factors underlying the health of a population. Prior studies have shown that neighborhood socioeconomic status, measured at the census tract-level, is associated with increased rates of heart disease, cancer, and all-cause mortality, even after individual-level socioeconomic status is taken into account [4-6]. Other studies show that zip codes in which children grow up influence their future income, educational attainment, fertility, and marriage outcomes [7]. Analyses of the impact of state Medicaid expansion status on comorbidity burden show the influence of state-level policy on health inequities [8].

Over the last decade, the availability of publicly accessible place-based data has greatly expanded [9-11]. However, the growth of data resources has been segmented and nonuniform. Many websites developed by individual governmental organizations or research groups offer domain-specific data, such as social environment, climate, health outcomes, or housing [12-15]. In addition, new or expanded place-based measures have been developed that are not yet accessible through online platforms [16,17]. For some domains (eg, rurality [18]), multiple measures exist but often are not available in one place to facilitate comparisons or assess suitability for particular applications. Furthermore, the availability of national data has become inconsistent and uncertain due to cuts to federal funding for biomedical research and shifting priorities at federal agencies [19-21]. The result is an extensive but evolving, complex, and fragile landscape of place-based measures.

UCSF Health Atlas [22] provides a single online platform for place-based data to help users explore place-based factors that influence health. It includes a curated repository of publicly available population estimates as well as expert-driven composite measures. Most data are available across all 50 states in the United States, the District of Columbia, and Puerto Rico.

The development of Health Atlas was initiated by the University of California, San Francisco (UCSF) Population Health and Health Equity program and led by the UCSF Population Health Data Initiative. The website was created in collaboration with Stamen Design, and its content was developed with expertise from the UCSF Department of Epidemiology and Biostatistics, UCSF Disparities Research Environment and Multi-omics Lab, and Plain Language Health. Health Atlas development began in 2019, and the initial California-only version was launched in April 2020 [23]. Over time, a mobile-enabled version, Census 2020 geographies, and new domains of data were added. In fall 2024, the Health Atlas team launched a national version that included national maps and data [24]. An artificial intelligence (AI)–assisted search function was integrated in January 2026.

## Data Assets

Health Atlas supports data visualization for 7 geographic levels: census tract, zip code tabulation area, county, congressional district, core-based statistical area, public use microdata area, and state. While not all data are available at every geographic level, most data are provided at the census tract level, the smallest unit of geography in Health Atlas (representing areas of approximately 4000 residents). Health Atlas provides data based on 2010 Census and 2020 Census boundaries; variables are merged on a geographic identifier, such as the Census Tract Federal Information Processing Series code [25], and transformed into flat files based on geographic granularity. Providing multiple census geographies allows researchers to select, download, and merge data relevant to their time period of interest.

To date, Health Atlas includes data on 206 variables (also referred to as “characteristics”) across 5 topical domains: demographics, socioeconomic, neighborhood, environment, and health and health care (Table 1). Domains were modeled after the Office of Disease Prevention and Health Promotion’s social determinants of health conceptual framework, which includes domains of health care access and quality, education access and quality, social and community context, economic stability, and neighborhood and built environment. Variables were selected for inclusion based on this framework, as well as feedback from expert scholars and potential users from academic, medical, and community settings. Data sources and variables are vetted for inclusion based on their relevance to place-based social determinants of health, accessibility (eg, from a public source), national scope, availability at the geographic boundaries represented in Health Atlas, and sustainability (eg, feasibility to update over time). Included data are curated primarily from public sources including the American Community Survey (ACS) [26] (eg, demographics, socioeconomics), US Centers for Disease Control and Prevention (CDC) PLACES: Local Data for Better Health [12] (eg, health and health care), and US Environmental Protection Agency (EPA) Environmental Justice Screen [27] (eg, environmental exposures).

**Table 1.** Health Atlas variable domains, subdomains, and sources (2025 version).^a^

Domain and subcategory	Sources
Demographic	
Age Sex Race and ethnicity	ACS^b^ 5-year estimates [28]
Socioeconomic	
Socioeconomic indices	Public Health Alliance of Southern California [29] Boston University: Institute for Equity in Child Opportunity & Healthy Development [30] CDC^c^: ATSDR^d^ [31] Custom measures generated by DREAM^e^ Lab [16] and HEAN^f^ [32]
Poverty and income	CDC PLACES [12] NCI^g^ [33] USDA^h^ Economic Research Service [34] HUD^i^ Comprehensive Housing Affordability Strategy [35] ACS 5-year estimates [28]
Income inequality Employment Education Digital access	ACS 5-year estimates [28]
Neighborhood	
Total population	ACS 5-year estimates [28]
Built environment	USDA Food Access Research Atlas [36] University of Michigan: NaNDA^j^ [37] EPA^k^ EJ^l^ Screen [27]
Housing	HUD Comprehensive Housing Affordability Strategy [35] ACS 5-Year Estimates [28] CDC PLACES [12]
Language and ethnic enclaves	Custom measures generated by UCSF^m^ DREAM Lab [16] ACS 5-Year Estimates [28]
Rurality	USDA Economic Research Service [34] US Census Bureau [38]
Segregation	Custom measures generated by HEAN [32] NCI [33]
Social environment	CDC PLACES [12] ACS 5-Year Estimates [28]
Structural racism	University of Michigan: Institute for Social Research [39]
Transit	CDC PLACES [12] ACS 5-Year Estimates [28] University of Michigan: NaNDA [37] EPA EJ Screen [27]
Environment	
Air pollution	EPA EJ Screen [27] Custom measures generated by UC^n^ CCHE^o^ [17]
Heat	Custom measures generated by UC CCHE [17]
Extreme precipitation	Custom measures generated by UC CCHE [17]
Environmental indices	EPA Public Health and Environmental Systems Division [40] CDC: ATSDR [31] FEMA^p^ [13]
Health and Healthcare	
Health outcomes	CDC PLACES [12] CDC NVSS^q^ [41]
Health care access	ACS 5-year estimates [28]
Health care use	CDC PLACES [12]
Health behaviors	CDC PLACES [12]
Disability	ACS 5-year estimates [28] CDC PLACES [12]
Mental health	CDC PLACES [12]

a Additional details for each variable are provided in MultimediaAppendices 1  2.

b ACS: American Community Survey.

c CDC: US Centers for Disease Control and Prevention.

d ATSDR: Agency for Toxic Substances and Disease Registry.

e DREAM: Disparities Research Environment and Multi-omics.

f HEAN: Health Excellence Action Network.

g NCI: National Cancer Institute.

h USDA: US Department of Agriculture.

i HUD: US Department of Housing and Urban Development.

j NaNDA: National Neighborhood Data Archive.

k EPA: US Environmental Protection Agency.

l EJ: Environmental Justice.

m UCSF: University of California, San Francisco.

n UC: University of California.

o CCHE: Center for Climate, Health, and Equity.

p FEMA: Federal Emergency Management Agency.

q NVSS: National Vital Statistics System.

The data are selected based on the most recent version available for a given geographic boundary. For example, the 2015‐2019 ACS variables (eg, for race/ethnicity) are available for Census 2010 geographies because they are the most recent variables available for that geography; the Census 2020 boundary is currently associated with the 2019‐2023 ACS variables. Health Atlas aims to update data annually following the release of new data from the Census and to search for additional updates to other variables at the same time. Health Atlas does not maintain longitudinal data, so when new data are released at an existing geographic boundary, it replaces the old version.

Full descriptions, data sources, available years, and geographies for each variable are provided on the Health Atlas and in MultimediaAppendices 1  2.

## Variable Types

### American Community Survey Variables

Five-year estimates from the ACS are downloaded from the tidycensus application programming interface package in R through a batch download. The Health Atlas includes many variables directly generated by and reported from the ACS. For example, “Overcrowding” is pulled directly from the Selected Housing Characteristics table. Others, such as “Seniors living alone,” are calculated internally based on ACS estimates:


$$\frac{Male\;householder\;over\;65\;living\;alone+Female\;householder\;over\;65\;living\;alone}{Total\;population\;65\;years\;and\;over}$$

### Data From Government Sites

Other publicly available variables, such as those from the EPA Environmental Justice Screen and CDC PLACES, are downloaded directly as .csv files from government websites.

### Index Variables

Health Atlas also includes selected index variables generated for Health Atlas by the UCSF Disparities Research Environment and Multi-omics Lab research group and the national Health Excellence Action Network. These include indices measuring socioeconomic status (ie, neighborhood socioeconomic status [nSES] [5], the neighborhood deprivation index [42], and racialized income segregation [also known as the Index of Concentration at the Extremes] [43]), indices measuring racial or ethnic segregation (ie, the local exposure and isolation index [LEx/Is] [44] and the location quotient [LQ]) [44], and indices measuring ethnic enclave status (ie, Asian American enclave and Hispanic enclave) [45,46]. These census tract-level indices are generated using ACS data and are presented as quintiles. Quintiles of nSES and ethnic enclave status are state-specific, based on the distribution of index scores in each state. All other indices are scaled to the United States.

### Historic Redlining

Historic redlining data were added to Health Atlas through a partnership with the UCSF Environmental Research and Translation for Health Center to highlight the effects of structural racism on neighborhoods tracing back to discriminatory home loan practices implemented by the Home Owners’ Loan Corporation (HOLC) in the 1930s. The HOLC created a ranking system for neighborhoods, now known as redlining, that categorized them by grade from A (“Best”) to D (“Hazardous”) based on data, including the quality of housing, recent values of sales and rentals, and the racial and ethnic composition of a neighborhood’s residents [29]. Health Atlas reports historical redlining using HOLC mortgage security risk maps that have been digitized by the Mapping Inequality Project [47] and overlaid with 2010 and 2020 Census tracts for selected US cities in ArcGIS by the University of Michigan, Institute for Social Research [48]. In Health Atlas, the color scheme from the original HOLC risk grades was implemented, where Green indicates Grade A, Blue indicates Grade B, Yellow indicates Grade C, and Red indicates Grade D.

### Climate Variables

The Health Atlas also includes multiple derived variables related to climate change in the state of California from the University of California Center for Climate, Health and Equity. Variables were derived using component data from the EPA Air Quality System and the Gridded Surface Meteorological data [49,50]. These data were added to contextualize air pollution, heat, and precipitation at the neighborhood level to inform local-level climate adaptation strategies. Example variables include average wildfire particulate matter 2.5, average heat index, number of days >95th percentile for maximum temperature, or maximum precipitation.

## Data Infrastructure

Data are partitioned by geographic level, census year, and variable and stored in Amazon Web Services S3 buckets, a public cloud storage resource [51]. This type of data organization (commonly called “hive partitioning”) is performed by using the DuckDB [52] and Polars [53] software packages. Storing the data statically reduces dependence on the server, making the platform more resilient.

Maps are generated using Mapbox [54] maps, which include data from OpenStreetMap [55]. Data descriptions are updated in Airtable [56], a cloud-based data storage platform, and mapped to the public-facing website. Metadata about each variable are loaded from Airtable into Health Atlas. When a user selects a variable and geography, the platform pulls only the relevant data from the S3 bucket and can load the values for the selection independently.

We aimed to meet Web Content Accessibility Guidelines level AA compliance for the interface with high text-to-background contrast ratios, minimum text sizes, and minimal decorative text. Maps and data visualizations are not strictly accounted for under Web Content Accessibility Guidelines, but we tested common points of concern, such as colorblind-friendly color palettes and figure-to-background ratios, to improve legibility during the design process.

An AI-assisted search function is integrated into Health Atlas to help users select their data of interest more efficiently. Suggestions are provided based on natural language processing via large language models and are limited to variables available in the Health Atlas.

## Tutorial

### Selecting a Variable

**Figure 1.**
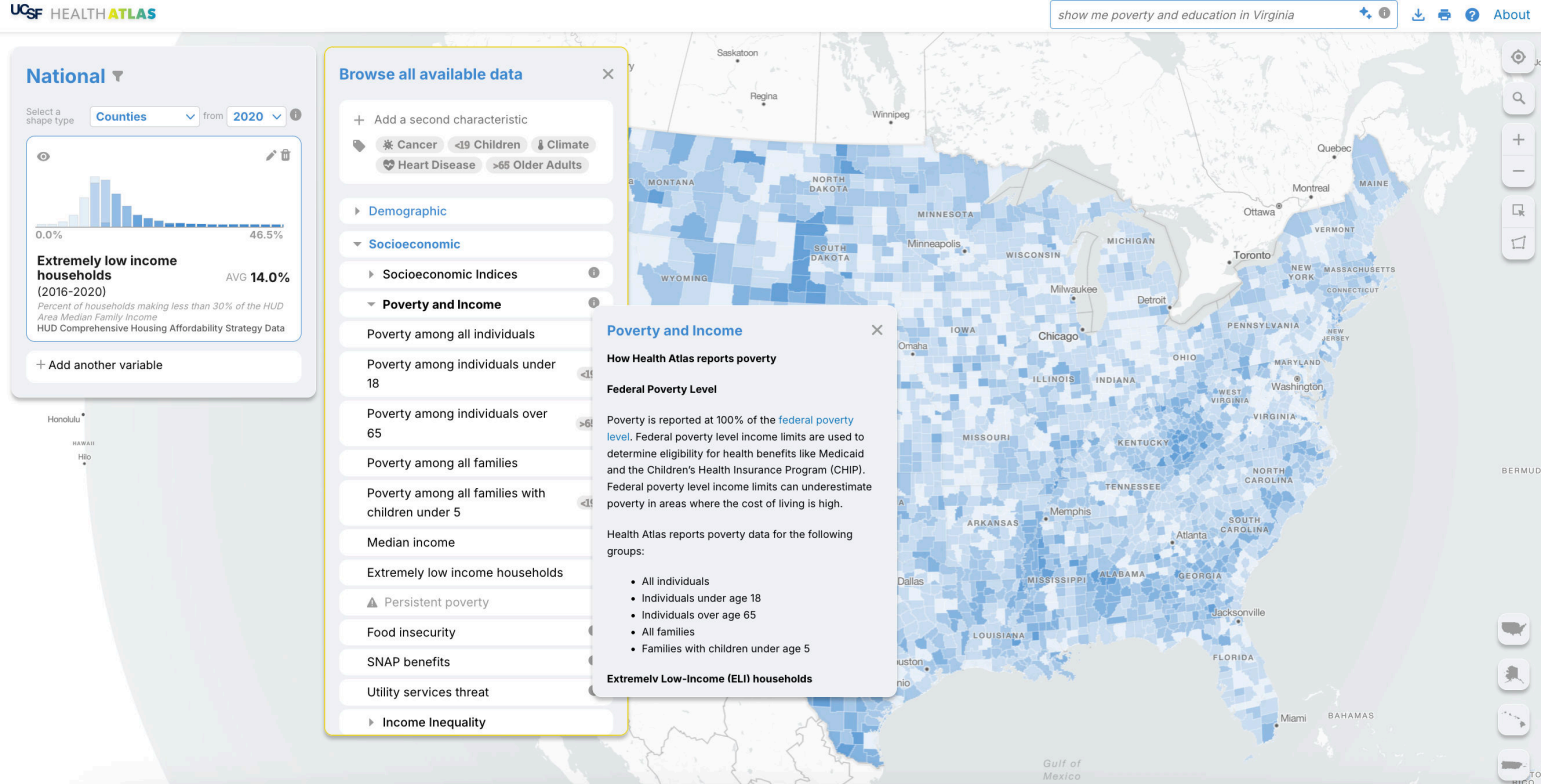
Health Atlas map showing the percentage of households that are extremely low-income across the United States at the 2020 Census tract-level. The selection panel shows a data description of Poverty and Income variables.

To select a variable, users can click on the name of the currently visible variable in the left-side panel. Another panel will pop up to browse all available data grouped by domain (demographic, socioeconomic, neighborhood, environment, or health and health care) and by tags (cancer, children, climate, heart disease, or older adults). If a variable is not available for a certain geography, it will be grayed out. If users attempt to select a geography or variable combination that is not valid, a pop-up panel will appear to provide an option to switch to the appropriate geographic boundary. Information on each variable, including source year, can be found in data descriptions by clicking the “i” icon next to the variable name. Figure 1 displays a pop-up window with the data description for Poverty and Income variables.

### Visualizing Data With Maps

**Figure 2.**
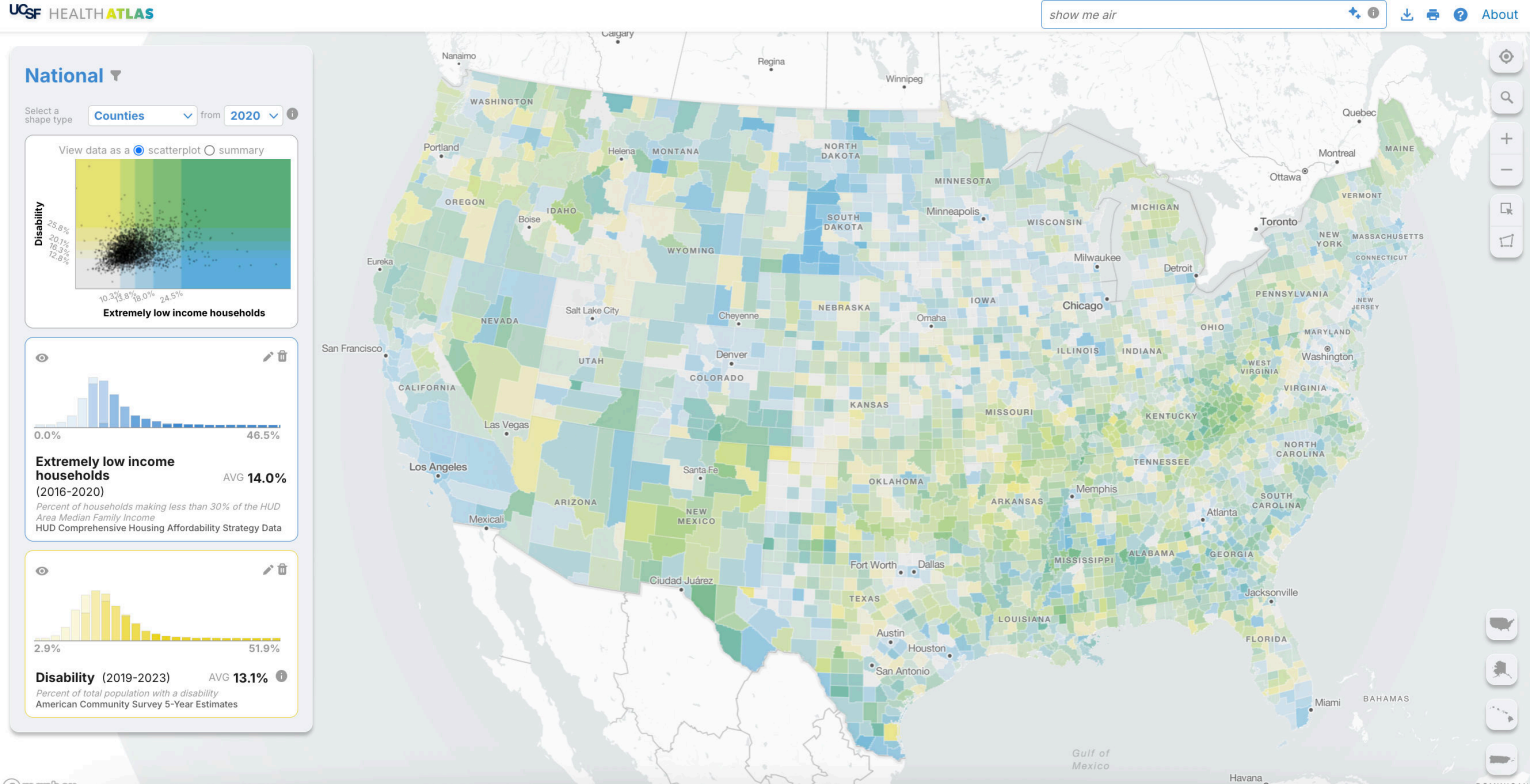
Health Atlas map of the percentage of households that are extremely low-income and the percentage of people with a disability across the United States at the 2020 Census tract-level.

Data are visualized in univariate and bivariate choropleth maps. Figure 2 displays the Health Atlas with 2 variables selected for 2020 census tracts: the percent of households that are extremely low-income and the percent of people with a disability. The color of the first variable (extremely low-income) ranges from light to dark blue, while the second variable (disability) ranges from light to dark yellow, and the data with the highest values for both are depicted in green. When zoomed out to approximately the continental United States, maps are displayed in an Albers projection; when zoomed into a state or county-level scale, maps are displayed in a Mercator projection. With inset maps in the bottom right corner of the main map, users can elect to view the contiguous United States, Alaska, Hawai‘i, or Puerto Rico. Users can easily navigate to noncontiguous areas (and back) via the insets.

### Using Histograms

**Figure 3.**
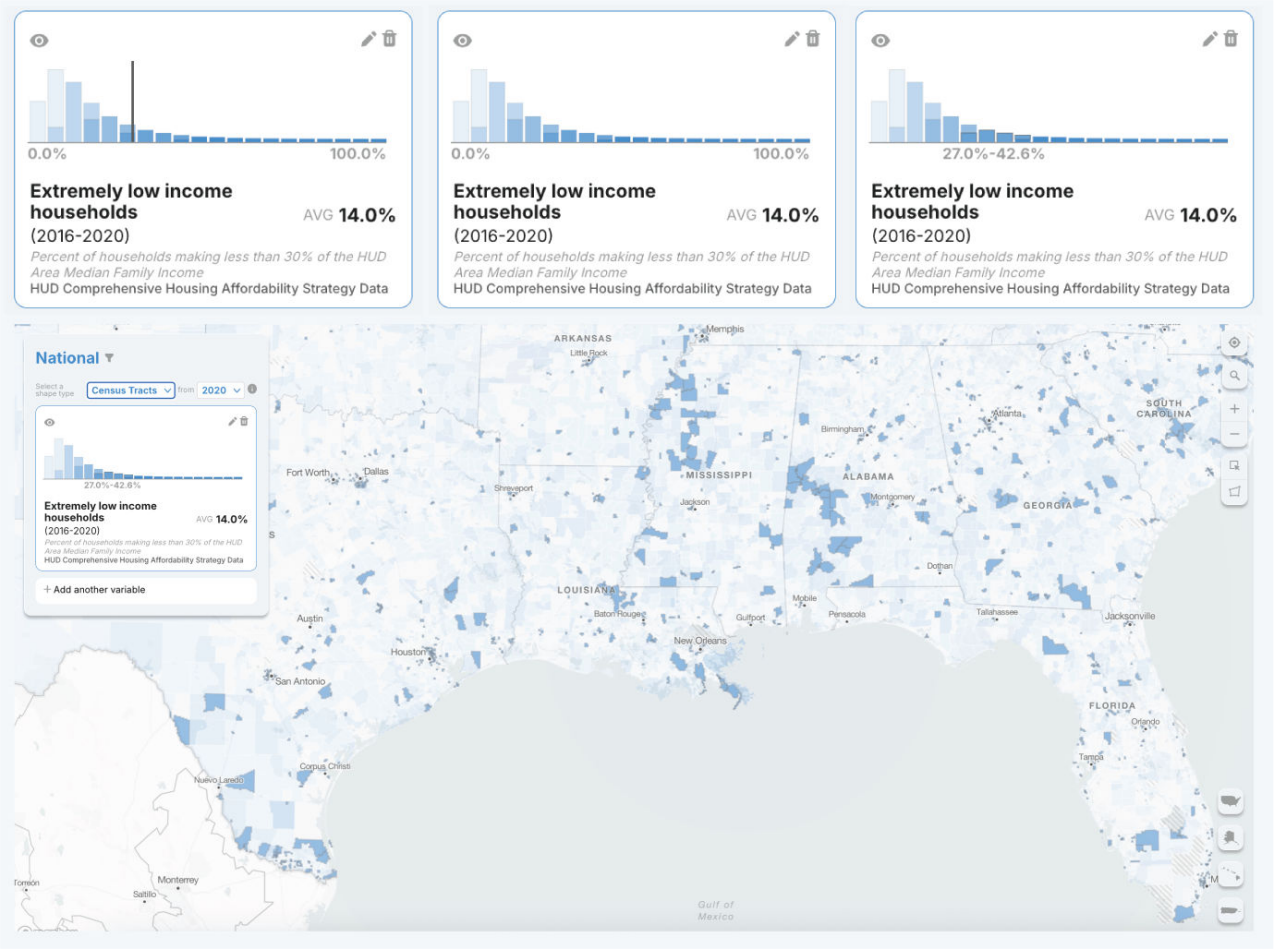
Three different states of a histogram on Health Atlas visualizing the distribution of census tracts according to the percentage of households that are extremely low-income. HUD: US Department of Housing and Urban Development.

Histograms act as a map legend and allow users to identify areas with similar values of the chosen variable; mousing over a geographic area shows where that unit lies in the overall distribution of the histogram by displaying a vertical line corresponding to variable values for that area (Figure 3). Histograms display data distributions using 20 bins for continuous variables, 5 bars for quintiles, 4 bars for some categorical variables (eg, rural or urban and historical redlining), and 2 bars for binary values. The map in Figure 3 shows how mousing over a choropleth bin in the histogram highlights geographic areas with values falling in that bin.

The bins of the histograms use equal intervals, but the colors of the bars follow natural breaks, which allow users to get a clear sense of the contours of the data. Natural breaks partition the data based on gaps in the data distribution [57] and are particularly effective for mapping variables with nonuniform distributions, which applies to most Health Atlas data from ACS, CDC PLACES, and other public sources. Choropleth bins are determined using *ckmeans* from the simple-statistics JS library by Tom MacWright [58]. A few Health Atlas variables are divided into equal intervals since they are displayed as quintiles (eg, nSES, ethnic enclaves) or scored as relative measures and ranked between 0 and 1 or 0 and 100 (eg, CDC Environmental Justice Index and Federal Emergency Management Agency National Risk Index, respectively).

### Using Scatterplots and Summary Plots

Like histograms, scatterplots serve as a map legend and a way to see the texture of the data. Scatterplots display how 2 variables relate to each other, with each dot representing a geographic unit (eg, each state, county, etc, depending on the selected level of aggregation). Scatterplots can help users descriptively visualize whether there is a linear, nonlinear, or null relationship between 2 selected variables on the map. Mousing over a geographic unit on the map outlines the point corresponding to that area on the scatterplot, so users can see the bivariate relationship of 2 selected variables for that geographic area.

**Figure 4.**
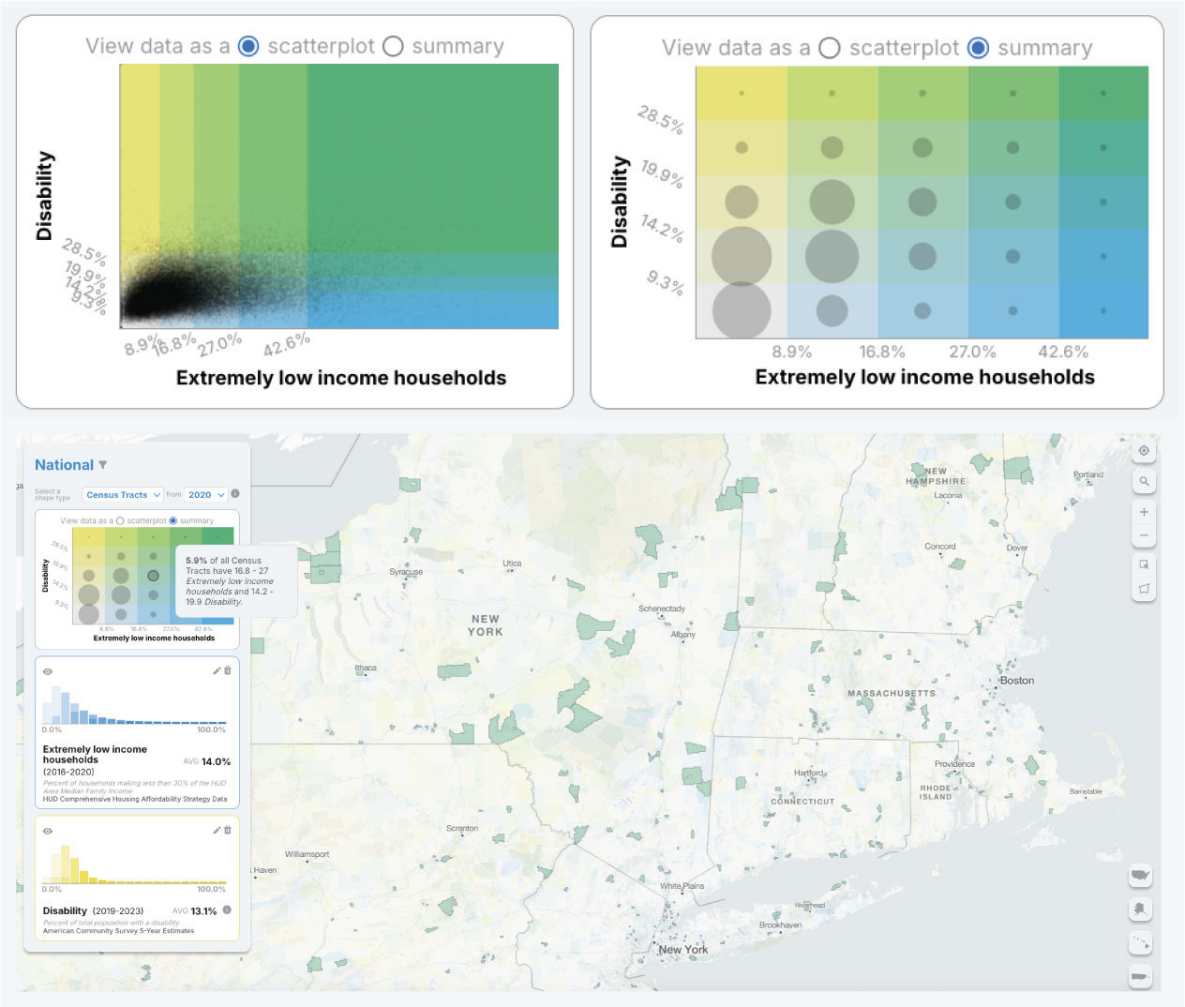
Bivariate distributions of extremely low-income households and disability across the United States at the 2020 Census tract level in the Health Atlas, using a scatterplot and summary plot.

The summary view is helpful for geographies such as census tracts, which generate thousands of points on the scatterplot. In addition, mousing over a bubble on the summary plot allows users to identify geographic areas that are similar in the relationship between 2 variables. Figure 4 shows that mousing over the center bubble on the summary plot highlights on the map the 5.9% of census tracts across the United States where 16.8% to 27% of households have extremely low income and 14.2% to 19.9% of residents have a disability.

### Custom Area Selection

**Figure 5.**
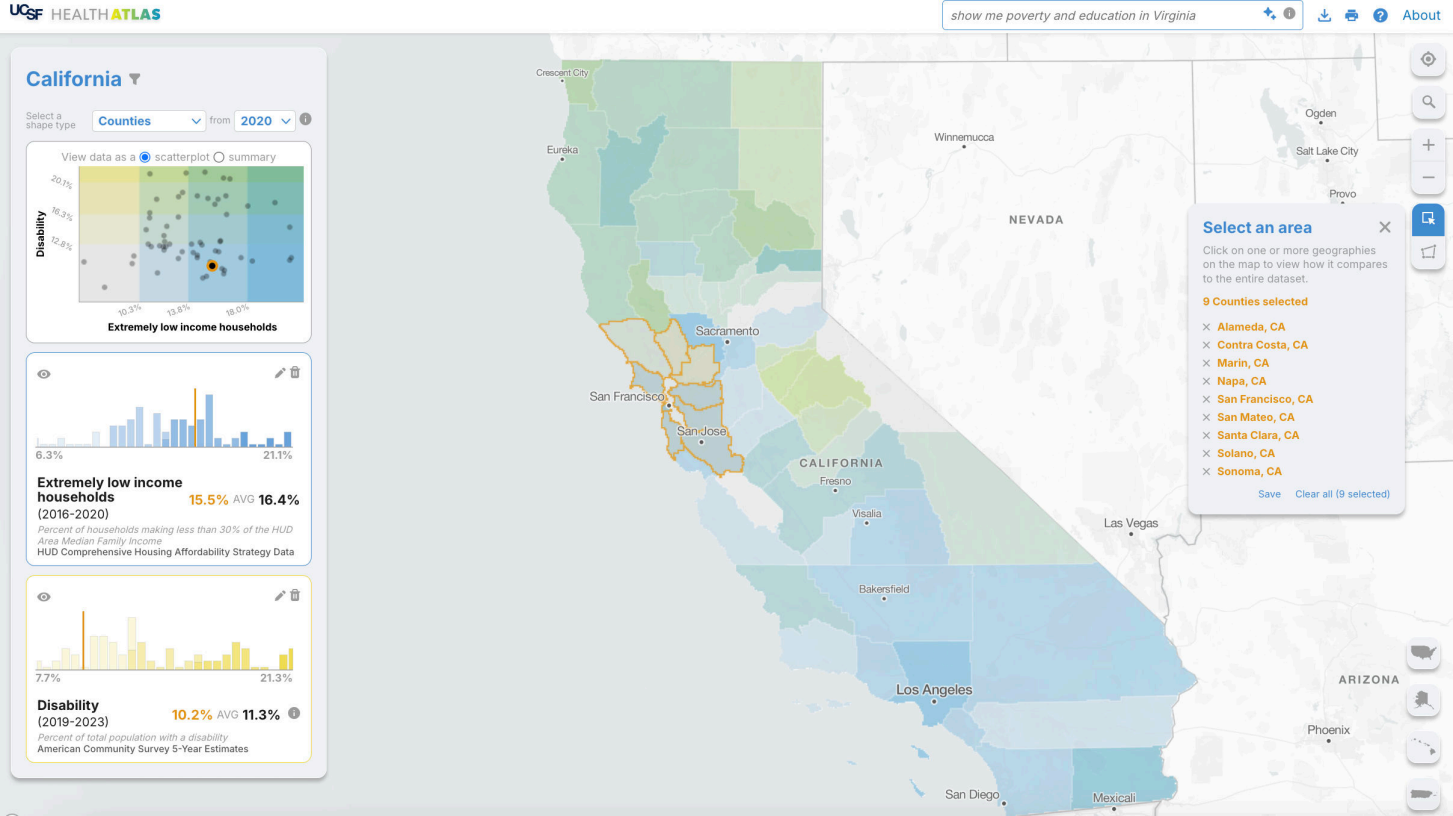
Health Atlas custom selection of 9 San Francisco Bay Area counties on a map filtered for the state of California showing the percentage of households that are extremely low-income and the percentage of residents that have a disability at the county level.

Users can select a custom area by using the “Select an area” or “Draw a shape” tools on the right side of the map. Once a custom area has been selected, weighted average values for the selected area are shown in orange in the histogram or histograms, scatterplot, and summary visualization. Figure 5 shows the selection of 9 San Francisco Bay Area counties outlined in orange on a map filtered for the state of California. The relative position of the bivariate relationship of the selected variables in the selected area is indicated with an orange point on the scatter and summary plots.

Raw data for population denominators at the census tract level are used to generate weighted averages for custom selection areas. For example, the value for “under 5 years old” for a joint selection of census tracts is calculated by summing each census tract value and dividing it by the total population. Meanwhile, the value for “poverty among individuals over 65” is calculated by summing each census tract value within the selected tracts and dividing it by the population size over 65. Some variables, such as indices or climate data, are not aggregated based on a population denominator. The Health Atlas data dictionary describes which population denominators, if applicable, are used to calculate weighted averages (Multimedia Appendix 1).

### AI-Assisted Search

**Figure 6.**
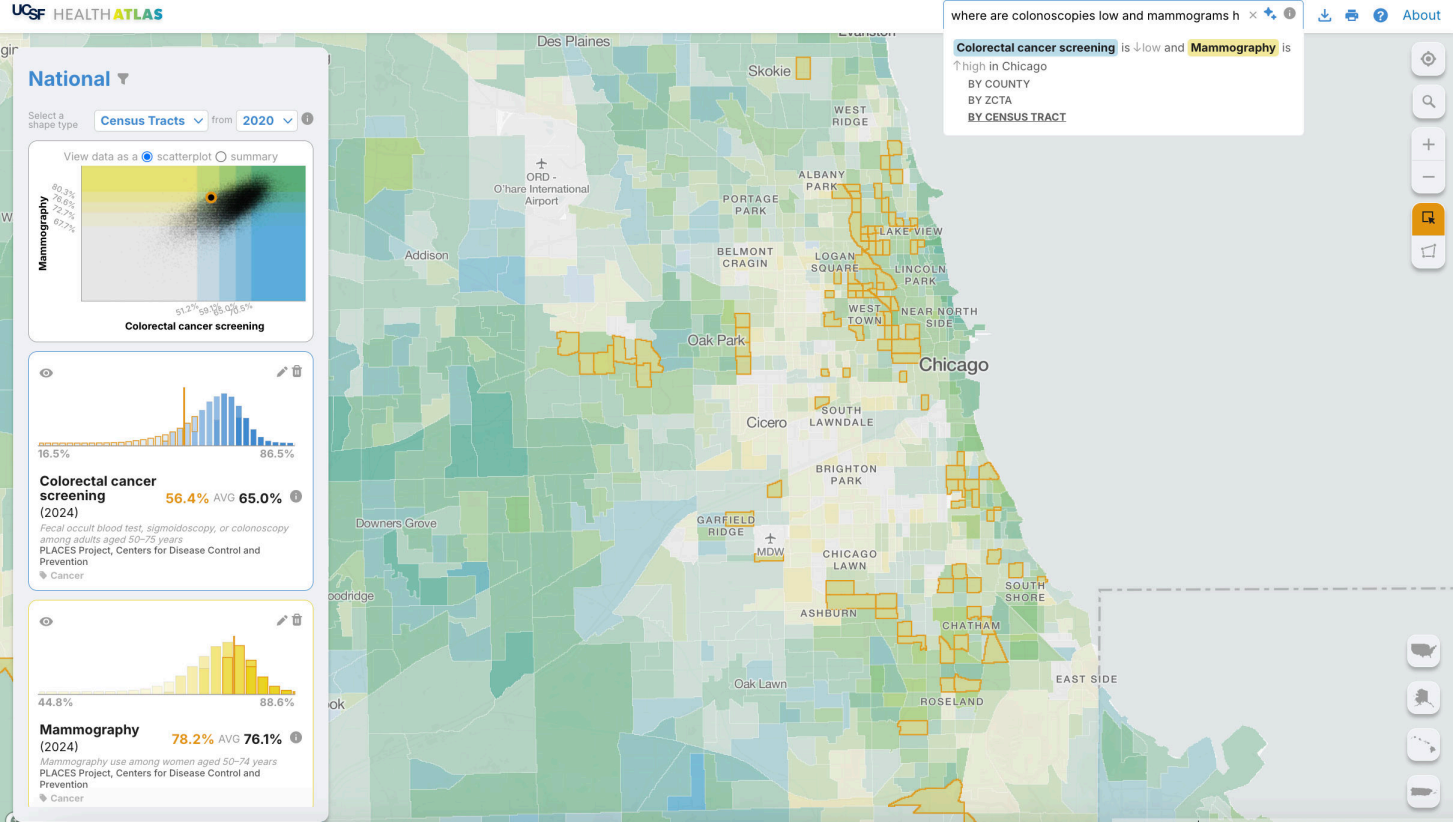
Health Atlas map of AI–assisted search identifying a map based on the query “Where are colonoscopies low and mammograms high in Chicago?”

Users can use the AI-assisted search function at the top of the website to guide variable selection. The search bar can process queries (eg, “show me poverty and education in Virginia”) and return variable and geography selections. The AI-assisted search function can also help users to zoom to specific areas of the map, filter to specific states, view variable suggestions, select available geographic boundaries, and identify regions with high or low values (Figure 6).

### Data Export

**Figure 7.**
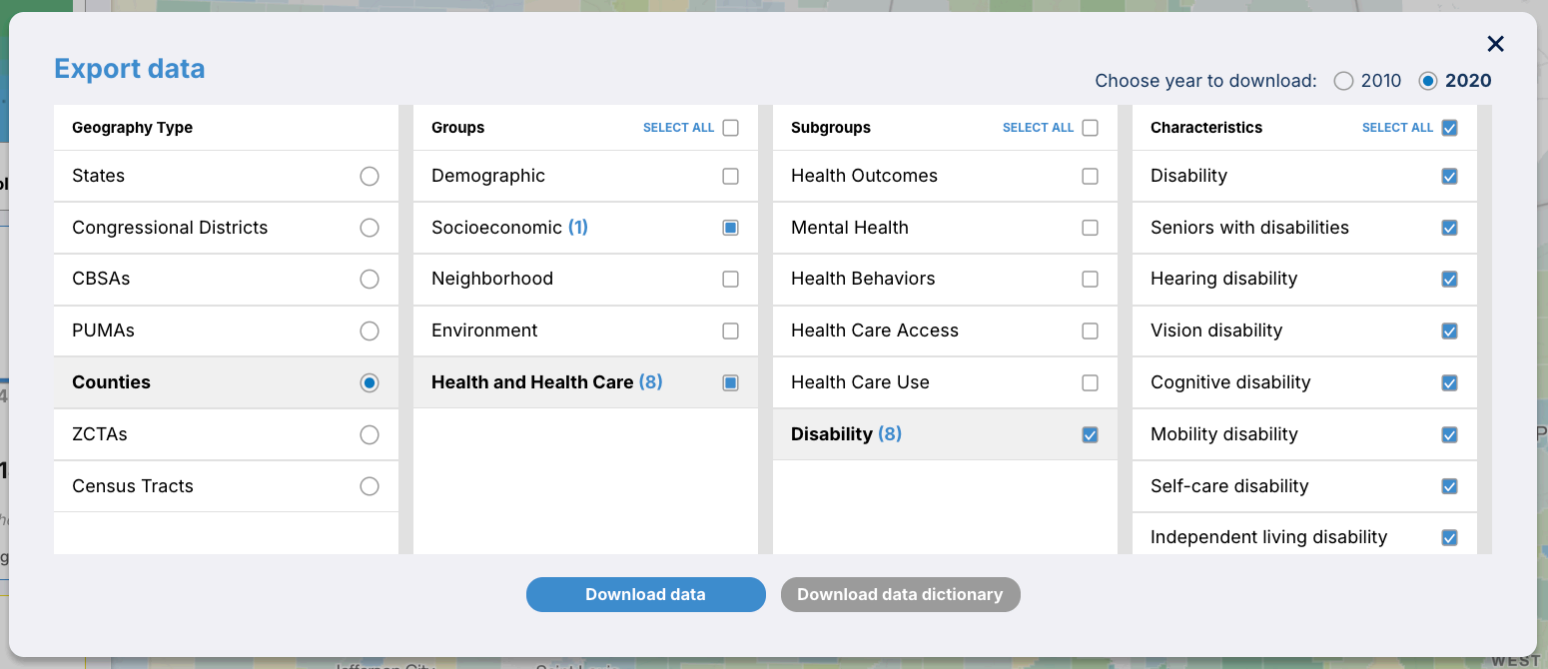
Health Atlas custom data download for socioeconomic variables and health and health care variables at the county-level. CBSA: core-based statistical area; ZCTA: zip code tabulation area.

Users can download Health Atlas data by clicking the download icon at the top right corner of the website. Data downloads will reflect the current parameters on the map and can be adjusted before download. Custom data exports allow users to download all data, or alternatively, data for a selected area and selected variables. Figure 7 shows an example of 1 user’s specifications to download data for a custom area selection for 10 socioeconomic variables and 8 health and health care (disability) variables at the county-level. The full data dictionary is also available for download.

Data are provided with geographic identifiers, facilitating the merging of exported data with study-specific datasets. A data dictionary is also available for download and includes the following information for each variable: name, brief description, categorization, units (eg, percent or quintile), data source, denominator for aggregation (if applicable), and available geographic units (MultimediaAppendices 1  2). Maps and visualizations can be exported as .png images for integration into presentations, reports, or proposals.

## Discussion

Health Atlas is a publicly available resource with over 200 place-based variables that can support a wide array of users interested in public health. Researchers can download data for analysis and maps for presentations or reports. Policymakers can gain insights into custom regions of interest. Community members can visualize local health and environmental factors for advocacy. Educators and learners can use mapping features to explore a variety of topics. By including national-level data, Health Atlas expands opportunities for collaboration by facilitating multistate comparisons and partnerships. Furthermore, it provides access to data that have not been readily available previously, such as segregation and ethnic enclave measures.

Health Atlas is aligned with contemporary conceptual frameworks that emphasize that health inequities largely stem from structural and social determinants of health across multiple levels of influence, including place [1,59]. Data hosted on Health Atlas, therefore, include measures of structural and social determinants of health across domains, such as the built and social environment, environmental and climate stressors, and health care access and use.

Health Atlas provides place-based variables across multiple domains free of cost to the user, curated from publicly available sources and/or research groups that collectively have not been previously collated or easily accessed (Table 1) [16,17,32]. It also provides comprehensive data downloads and data visualizations across domains and geographic units (eg, census tract, county, state). Providing granular levels of data, such as census tracts, is key to meaningfully capturing neighborhood-level exposures. Providing data at multiple levels, including nonnested geographic units such as congressional districts and public use microdata areas, provides users the flexibility to use the data for different applications such as advocacy, policy, and administration. Other resources, such as PolicyMap [60] and SocialExplorer [61], include a wider set of similar data but require institutional licenses for access to the data, whereas Health Atlas data are publicly available. Tools such as ArcGIS [62] can also provide maps with more sophisticated features, including spatial analysis or the ability to layer study data in a secure environment; however, access to these tools typically requires licenses. While some licensed access additionally allows for a platform in which users can upload and incorporate their own data into maps, Health Atlas allows users to download a customizable dataset to append to their data.

The ability to gain insights from data is key and should not be limited to those with advanced training. Health Atlas provides easy-to-access maps, histograms, scatterplots, and summary plots to allow users to better understand the distribution of the data. Data descriptions and links to original sources are included for each variable. Through partnership development, Health Atlas makes new measures available to the public (eg, segregation, climate). For these measures, more detailed descriptions of how variables were created are provided, with citations to relevant published articles.

Health Atlas has remained nimble in response to user input, new partnerships, and changing external circumstances. The site has evolved to include tags for filtering variables related to selected topics, additional variable domains and categories, and a function to easily export map images. The Health Atlas scientific team also has back-end capabilities to update data and data descriptions as needed.

Since Health Atlas was first released in April 2020, it has been used for a variety of public health applications. During the COVID-19 pandemic, Health Atlas allowed users to visualize COVID-19 data alongside place-based data to make evidence-based decisions for organizations and individuals [63]. It also helped researchers and policymakers better understand how different communities were being impacted by the pandemic, and how social factors, such as neighborhood-level English proficiency and disability, contributed to disparities in COVID-19 outcomes [64,65]. The University of California Davis Comprehensive Cancer Center is currently using Health Atlas to identify areas with low breast cancer screening to plan locations for their mobile mammogram bus program (J. Dang, personal communication, October 30, 2025).

Health Atlas has also been used to link place-based data to geocoded patient addresses from individual-level datasets or electronic health records, providing insight into multilevel determinants of health. At UCSF, a project linking nSES from Health Atlas to electronic health records data found that older adults living in low SES neighborhoods had lower odds of advance care planning [66], a process by which people communicate their preferences for future medical care. Another project used nSES from Health Atlas to adjust for confounding at the neighborhood level and provide a clearer picture of the direct benefits of a digital health intervention among patients participating in cardiac rehabilitation [67]. Additional research used geospatial methods to plot hotspots of patients with serious mental illness and characterize neighborhoods based on racial or ethnic composition and socioeconomic status using data accessed through Health Atlas [68]. These examples of multilevel research studies demonstrate the intersection of individual and place-based factors and provide context and nuance for health professionals and policymakers to better address structural and social determinants of health.

While Health Atlas can help to quickly visualize geographic differences within the framework of an ecological study, users should avoid attributing place-based factors or associations to individuals residing within those places (ie, ecological fallacy) [69]. The interpretation of descriptive results should include consideration of the time periods over which measures were derived (source years) and possible confounding factors. Moreover, place-based data cannot replace local expertise on how residents interact with places and how these various attributes influence their health.

Data available through Health Atlas are pulled primarily from publicly available sources. If the reliability and availability of emerging data change, the tool may not accurately represent the population and the places where they reside. In some cases, data were not available for certain areas or years (eg, PLACES Health-Related Social Needs, which were not derived for all states by the CDC) not relevant for certain regions (eg, ethnic enclaves, which were only generated for states with sufficient representation of Asian American or Hispanic residents), or were not generated for all states due to the complexity of the data (eg, modeled climate data such as heat). Furthermore, the use of geographic boundaries such as census tracts can limit data presentation and analysis for Health Atlas [59]. While the availability of multiple geographic units allows for flexibility, the results of an analysis may be dependent on the size, shape, or orientation of the chosen geographic unit (ie, Modifiable Areal Unit Problem) [70].

Health Atlas is a scientifically robust data repository that represents an important contribution to the landscape of place-based data available to the public. We envision that the national Health Atlas will contribute to evidence-based community-based initiatives, impactful health equity research, and effective public health programs. Future directions for Health Atlas include further integration of AI, improved accessibility, incorporation of longitudinal data, and automated reports.

## Supplementary material

10.2196/89065 Multimedia Appendix 1 Health Atlas data dictionary variable categories, subcategories, names, denominators, units, and sources.

10.2196/89065 Multimedia Appendix 2 Health Atlas data dictionary variable categories, subcategories, names, export availability, geographies, and Census years.

## Abbreviations

ACS: American Community Survey
AI: artificial intelligence
CDC: US Centers for Disease Control and Prevention
EPA: US Environmental Protection Agency
HOLC: Home Owners’ Loan Corporation
NCI: National Cancer Institute
nSES: neighborhood socioeconomic status
UCSF: University of California, San Francisco
